# Effects of Bending Load Level and Cementitious Capillary Crystalline Waterproofing Content on Chloride Transportation in Jointed Concrete

**DOI:** 10.3390/ma19102069

**Published:** 2026-05-15

**Authors:** Yongdong Yan, Daniel Mishael, Chunhua Lu, Lei Tan

**Affiliations:** Department of Civil Engineering, Jiangsu University, Zhenjiang 212000, China; yand@ujs.edu.cn (Y.Y.); lch79@ujs.edu.cn (C.L.); tanlei0217@163.com (L.T.)

**Keywords:** jointed concrete, bending load, chloride ion, cementitious capillary crystalline waterproofing material

## Abstract

The composition and interface quality of jointed concrete can significantly influence chloride ion penetration, especially in coastal environments. This study investigates the transport behavior of chloride ions in concrete flexural members with varying joint configurations—no joint, smooth wet joint, and roughened wet joint—under different bending loads. After 28 days of curing, specimens were subjected to bending loads and immersed in an 8% NaCl solution for 300 days. Chloride ion concentrations were then measured at different depths and locations. Results revealed that joints, particularly smooth wet joints, significantly accelerate chloride ion transmission, and that chloride accumulation at the joint is consistently higher than in adjacent areas or jointless concrete. The apparent diffusion coefficient of chloride ions was notably higher at joint interfaces and increased with bending load level due to microcrack formation. Notably, the incorporation of Cementitious Capillary Crystalline Waterproofing (CCCW) in the concrete mix improved resistance to chloride ion penetration. A dosage of 1% CCCW proved most effective, reducing the diffusion coefficient at the joint by approximately 10%—demonstrating an optimal balance between performance and material efficiency. These findings provide practical guidance for improving the durability of jointed concrete structures in chloride-rich environments.

## 1. Introduction

Concrete joints are essential components in mass and prefabricated concrete constructions, playing a crucial role in the building process [1]. However, the differences in materials between joints and monolithic poured concrete may adversely influence the performance of concrete structures, particularly in coastal environments where chloride ions are prevalent [2,3,4]. Such joints are particularly susceptible to accelerated chloride ingress, potentially resulting in premature structural degradation. To ensure the durability and proper functioning of jointed concrete structures in chloride-rich environments, systematic research on their performance is essential.

For concrete structures with joints, the microstructure at the joints significantly impacts their durability. Zhang et al. [5], using scanning electron microscopy (SEM), discovered that cement hydration at joints was incomplete, revealing microcracks, larger pores, and lower density compared to the concrete matrix. He et al. [6] analyzed various concrete cross-sections, finding that the bonding performance between new and old concrete improved with increasing fractal dimensions at the interface. Roy et al. [7] emphasized that jointed concrete structures in coastal environments are susceptible to accelerated chloride ion ingress, making these joints critical areas for corrosion due to interactions with oxygen, water, and fluctuations in humidity and temperature. Angst [8] underscored how cracks, including those at construction joints, significantly affect the corrosion protection provided by the concrete cover, underscoring the necessity of addressing these vulnerabilities. Bassuoni and Nehdi examined the chemical resistance of self-consolidating concrete, noting the impact of joint interfaces on sulphuric acid attack resistance [9]. Jianzhong [10] emphasized the need for high-quality joint construction to improve overall durability in chloride-exposed environments. Additionally, Costa and Appleton [11] exhibited through case studies showing that concrete deterioration in marine environments is significantly influenced by the quality of the joints, emphasizing the importance of effective joint construction and maintenance.

The chloride ingress behavior of jointed concrete has been further investigated in various studies. Li et al. [12] conducted chloride ion corrosion tests on jointed concrete specimens, revealing that concrete with direct and chiseled wet joints exhibited lower resistance to chloride ion ingress than monolithic concrete. Huang et al. [13] found that chloride-induced corrosion at construction joints was consistently more severe than in non-jointed areas, particularly in cyclic wet–dry environments. This remained evident even for stainless steel reinforcement, although epoxy-coated steel bars were excluded from the study.

Yoo et al. [14] examined chloride transmission in cold joint concrete under pressure, showing that the chloride diffusion coefficient increased linearly with compressive load. Li et al. [15] found that appropriate compressive stress can reduce the negative effects of joints on chloride resistance, based on a salt spray test on joint concrete specimens under varying stress states. This suggests that appropriate compressive stress can reduce the negative impact of joints on concrete’s resistance to chloride ion erosion [16].

Environmental factors such as freeze–thaw cycles and wet–dry conditions also exacerbate chloride penetration in jointed concrete. Zhang et al. [17] demonstrated that freeze–thaw cycles significantly increase chloride diffusion in fly ash-mixed concrete, reducing its durability. Qayyum [18] reviewed the use of waste glass in cement and concrete production, highlighting its potential in improving durability against chloride ingress. Papadakis [19] examined the impact of supplementary cementing materials, including high volumes of fly ash, on the durability of concrete, noting enhanced resistance to chloride ingress. Chen et al. [20] emphasized the detrimental effects of cyclic wet–dry conditions on chloride resistance in concrete. Moreover, Neville [21] noted that lower water-to-cement ratios combined with supplementary cementitious materials, such as fly ash, enhance resistance to chloride penetration. The impact of environmental cycles on jointed concrete emphasizes the need for high-quality construction techniques and material selection to improve durability in chloride-exposed environments. Aldea [22] quantitatively compared methods to measure the permeability of cracked concrete, demonstrating that permeability substantially governs chloride ingress. Nabighods [23] examined the effect of cement type on the resistance of self-consolidating concrete against magnesium sulfate attack, noting that cement type also affects chloride resistance. Guneyisi et al. [24] investigated the mechanical behavior and durability of self-compacting concrete with high volumes of supplementary cementitious materials, finding improved durability against chloride attack. Gmawlue et al. [25] emphasized the need for high-performance concrete for durable marine infrastructure, particularly in chloride-rich environments. Research has investigated the effects of time and cold joints on chloride diffusion in concrete containing Ground Granulated Blast Furnace Slag (GGBFS) under various loading conditions. The findings highlight that monolithic cast concrete has superior resistance to chloride salt attack compared to cold joint concrete. However, accurately determining the complex strength and damage behavior of the concrete, especially considering uncertainties such as sulfate exposure and variations in cold joint surfaces, presents challenges when applying classical elastic-plastic theory and damage codes.

In addition to chloride ingress, sulfate attack is also a major concern for jointed concrete in coastal environments. Qin et al. [26] studied cold joint concrete under sulfate dry–wet cycles with loading and found that the strength, elastic strain, and dissipation energies fluctuated with the number of cycles. These findings indicate that alternating weather conditions can exacerbate the degradation of concrete joints, leading to a reduced service life.

Recent research has explored repair and protection strategies for concrete joints in chloride-rich environments. Self-healing techniques (Xu et al. [27]), chloride-induced reinforcement corrosion behavior in self-healing concrete [28], and corrosion inhibitors [20] have all been examined as potential solutions. While these studies provide valuable insights, there remains a need for more effective strategies to enhance the durability of concrete joints in coastal areas.

**Figure 1:**
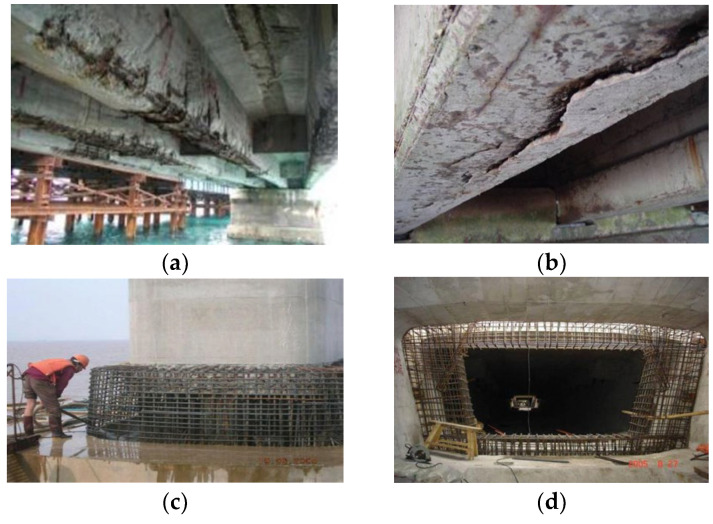
Wet joint of cracking of cross-sea bridge concrete caused by chloride erosion (**a**,**b**); (**c**) wet joint between pier and platform; (**d**) wet joint between box girders.

However, in coastal structures, construction joints particularly at column-to-deck interfaces or between box girders are prone to accelerated chloride attack under tidal spray and cyclic loading. Figure 1a,b show typical “wet joints” on cross-sea bridge cracks initiated by chloride erosion at a pier deck interface; (c) shows the joint between a pier and platform; and (d) shows a joint between precast box girders. These degraded field joints relate with the specimens whose geometry and joint details are illustrated in Figure 3. In Figure 3a, the jointless specimen PCW represents a monolithic section with no intentional interface. Figure 3b shows the smooth wet-joint configuration P1-C0-S as a PVC sheet simulating a planar casting interface at the side of the beam, mimicking the flat contact surfaces seen in Figure 1c. By matching the field joint types (smooth vs. rough) and their location in a bending member, the numbering convention (for example P1-C1-S for 0.6 Pcr, 1 % CCCW, smooth joint) directly links each lab specimen back to the real-world scenario shown in Figure 1. This ensures that the chloride-ingress tests under a sustained flexural load represented a reproduction of the critical features of coastal concrete joints.

Therefore, this study proposes the use of cementitious capillary crystalline waterproofing as a more effective solution to address chloride ion attacks in coastal environments. To further investigate the impact of bending loads and joint types on chloride ingress, this research designs concrete members with different joint configurations, applies bending loads, and conducts chloride erosion tests. By analyzing chloride ion concentrations near the joints, the study aims to enhance our understanding of concrete durability in coastal environments and provide practical insights for improving joint performance.

## 2. Materials and Experimental Method

### 2.1. Material

For this experiment, Jiangsu Helin P.O 42.5 cement was used, with its chemical composition detailed in Table 1. River sand, with a fineness modulus of 2.6, served as the fine aggregate, while 5–10 mm continuous graded crushed stone was chosen as the coarse aggregate for its superior interlocking properties. Cementitious Capillary Crystalline Waterproofing (CCCW), provided by Beijing Chengrong Company (Beijing, China), was added to enhance resistance to chloride ingress. A polycarboxylate high-efficiency superplasticizer improved the workability of the mix, maintaining a low water-to-cement ratio critical for durability. Together, these materials aimed to produce a concrete mix that is both mechanically robust and resistant to chloride-induced corrosion.

The mix ratios of concrete are shown in Table 2 below. Four kinds of CCCW contents, represented as C0, C1, C2, and C3, respectively, were considered in the experiment, as seen in Table 2.

### 2.2. Specimen Preparation

Two HRB 400 steel bars with a 12 mm diameter (*GB 1499.2–2018*) were placed at the bottom and top of the specimen to provide tensile reinforcement. As there was no shear force acting within the member during the loading process, no stirrups in the middle were included. However, two HPB 300 round steel bars, each 8mm in diameter, were positioned at both ends of the specimen to form a steel skeleton. This configuration helped stabilize the structure, as visually represented in Figure 1 [31,32], ensuring adequate support without unnecessary reinforcement in the shear-free zones [33].

**Figure 2:**
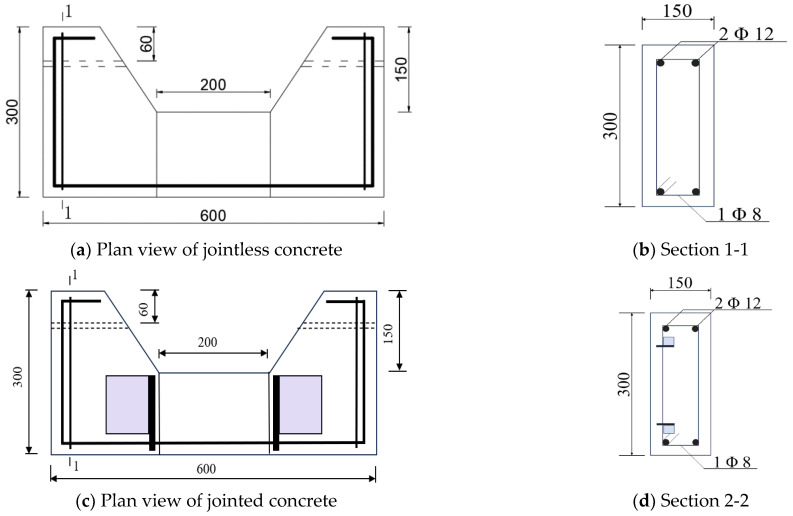
Section details of test specimen.

To construct the sample specimen, two brackets were designed at each end, each with a 10mm diameter hole aligned longitudinally to facilitate the application of long-term loads (see Figure 2). The force-bearing section in the middle measures 200 mm in length, with a cross-sectional dimension of 150 mm × 150 mm [33]. In addition to varying CCCW content, the experiment also examined the effects of joint form and bending load levels. Joint forms were categorized as no joint (W), smooth wet joint (S), and roughened wet joint (Z) [34]. The bending load levels were set to 0, 0.6P, and P (denoted as P0, P1, and P2), where P represents the load at which the specimen cracks. The parameters and specimen numbers are detailed in Table 3 [35,36,37].

**Figure 3:**
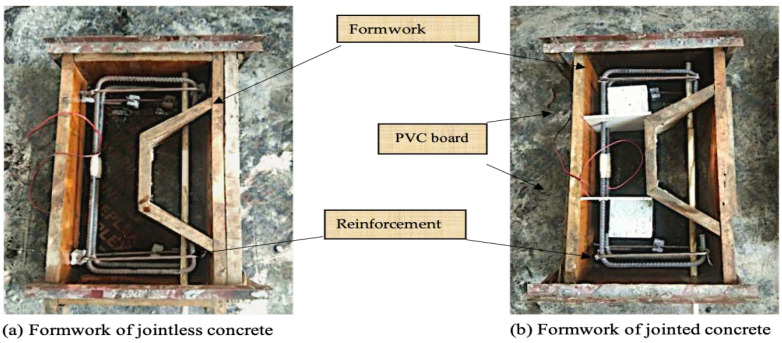
Formwork arrangement of different concrete specimens.

The specimen numbering convention combines the load level, CCCW content, and joint form. For instance, P1-C1-S denotes a load level of 0.6P, a CCCW content of 1%, and a smooth wet joint. To control the load during testing, strain gauges were attached to the center of the tensile reinforcement and protected with epoxy resin. The reinforcement skeleton was then placed in the formwork, with a 10mm PVC pipe inserted into the reserved hole. For jointless specimens, concrete was cast directly into the formwork (see Figure 3a). In jointed specimens, since joints are typically located in low-stress areas in real engineering scenarios, they were positioned at the sides of the specimen instead of mid-span. Plastic sheets were placed on the right section of the formwork (see Figure 3b), and concrete was first cast in the central part of the component. After 24 h, the plastic sheets were removed, and concrete was cast on the sides. For roughened wet joint specimens, the pre-cast concrete interface was manually chiseled to an average depth of 2 mm before casting the concrete on the sides. After 24 more hours, the formwork was removed, the PVC pipe was extracted, and the specimen was cured in an environment with 95% relative humidity at 20 °C for 28 days.

### 2.3. Experiment Method

A high-strength threaded rod with a diameter of 10mm was inserted into the reserved hole of the specimen, and bending load was applied by turning the nuts at both ends of the rod. During the loading process, strain on the main tensile tendons was measured in real time to control the loading value. Initially, load was applied to the specimen P2-C0-S until tension cracks appeared. At this point, the steel strain was recorded, and the width of the concrete cracks was measured. The load at which the cracks occurred was noted as the cracking load, Pcr. Steel strain corresponding to 0.6 Pcr was then converted accordingly. The same procedure was used to apply load to the remaining specimens, adjusting the steel bar strain to achieve the designed load levels. The actual load levels and mid-span crack widths for each specimen are detailed in Table 4.

**Figure 4:**
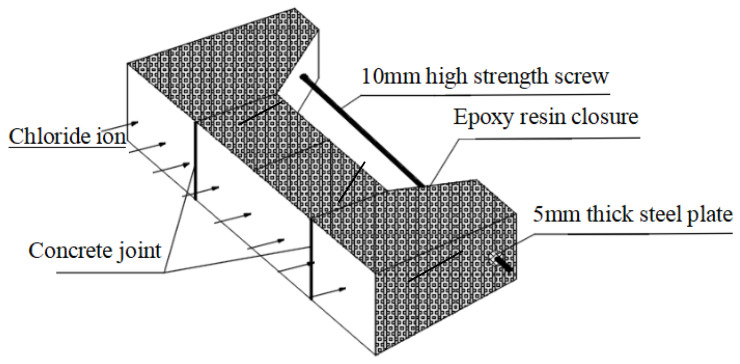
Loading and exposure condition of concrete specimen.

After applying the load to the specimen, the tension surface was exposed while the non-exposed surfaces, including the screws and nuts, were sealed with epoxy resin. Once the epoxy resin had fully cured, the specimens were immersed in an 8% NaCl solution for chloride corrosion testing. The exposure conditions for the loaded concrete specimens are illustrated in Figure 4.

**Figure 5:**
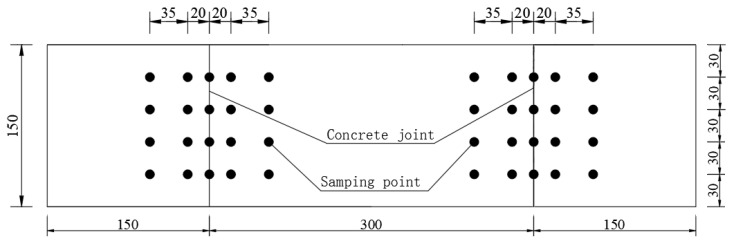
Distribution of sampling point position (mm).

After 300 days of immersion, the specimens were removed from the solution and allowed to air-dry for seven days. Concrete powder samples were then collected from various depths of the exposed surface, and chloride ion concentrations were measured for each sample. The potential of each sample solution was tested using a Soaked RCT [1] electrode, and the measured potentials were converted to free chloride ion concentrations based on pre-calibrated results. Figure 5 illustrates the distribution of the sampling points.

### 2.4. Test Matrix and Statistical Analysis

For each test condition, three identical specimens were prepared and subjected to the same testing protocol to ensure the reproducibility and reliability of results. The chloride ion concentration and the apparent diffusion coefficient values reported in this study represent the average of these three replicates. Additionally, standard deviation and coefficient of variation (CoV) were computed for each condition to evaluate data variability and ensure statistical robustness. These values have been considered in the interpretation and discussion of the results to enhance the credibility of the findings as discussed below.

## 3. Results and Discussion

### 3.1. Effect of Joint Type on Chloride Ingression in Loaded Concrete Members

The initial phase of the experiment assessed how different joint types affect chloride ingression. Concrete members exhibited varying levels of chloride ion concentration depending on joint type and immersion depth.

#### 3.1.1. Lateral Distribution of Chloride Ion Concentration 

**Figure 6:**
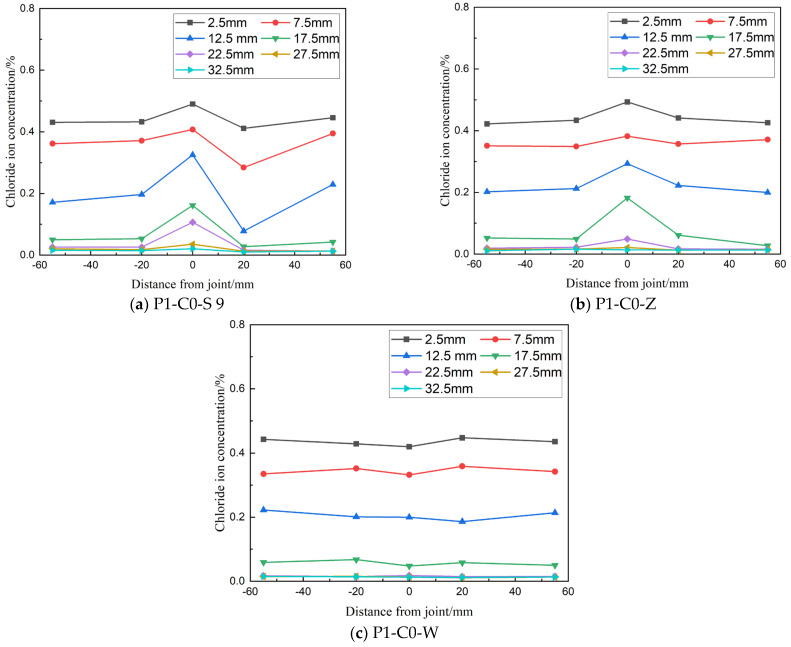
Lateral profiles of free chloride concentration at smooth wet joint under 0.6 P-cr (28 d cure, 300 d immersion).

Figure 6 illustrates the lateral distribution of chloride ion concentration at different depths in concrete members with joints under a detected load level of 0.6 Pcr. Chloride ion concentration consistently decreases with increasing depth in all specimens. For specimens with direct wet joints (P1-C0-S) and chiseled wet joints (P1-C0-Z), the chloride ion concentration at the joint is higher compared to both sides of the joint, indicating that these joint types negatively impact the concrete's resistance to chloride ion erosion. Additionally, at a load level of 0.6 Pcr, chloride ion concentrations measured at 20 mm and 55 mm from the joint are nearly identical, suggesting that this load level has a minimal effect on the rate of chloride ion transmission in the concrete tension zone.

**Figure 7:**
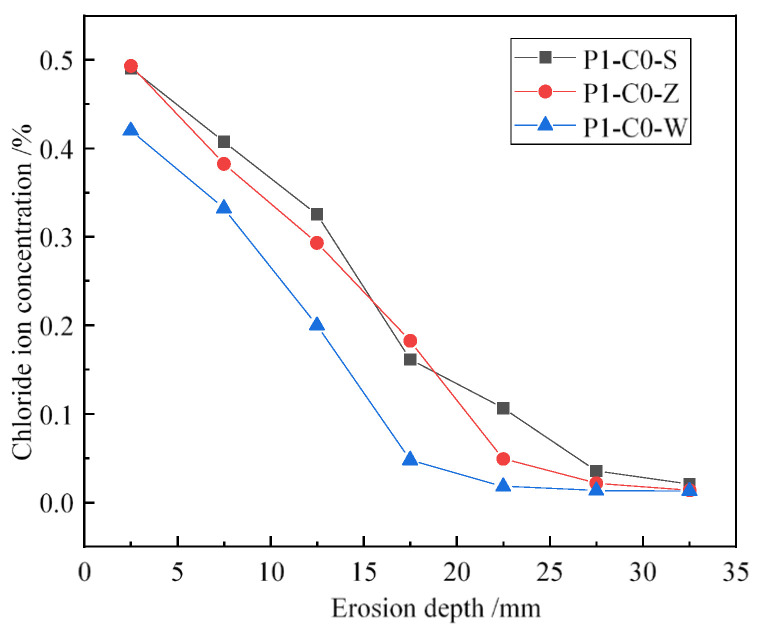
Chloride ion concentration at different types of joints.

To better understand the impact of different joint types on chloride ion penetration in flexural components, we analyzed chloride ion concentrations at joints with identical mix ratios (JZ) and load levels (P1), as illustrated in Figure 7. The results show that chloride ion concentrations at joints in flexural members with direct wet joints and chiseled wet joints are significantly higher than in jointless concrete specimens. Specifically, at a depth of 12.5 mm, chloride ion concentrations in components with direct wet joints and chiseled wet joints are 62.7% and 46.6% higher, respectively, compared to those in seamless concrete specimens. Roughening treatments at joints can effectively reduce chloride ion concentrations compared to direct wet joints. At depths of 7.5 mm and 12.5 mm, concrete specimens with roughened wet joints exhibit reductions in chloride ion concentrations of 6.2% and 9.9%, respectively, compared to those with direct wet joints.

#### 3.1.2. Joint Treatment on Apparent Chloride Ion Diffusion Coefficient

Chloride ions in concrete immersed in a chloride salt solution primarily move via diffusion driven by concentration gradients [33,39,40,41]. This diffusion process is described by Fick’s second law, which accounts for the movement of chloride ions in both free and bound forms within the concrete. The one-dimensional diffusion equation for chloride ions can be expressed as follows:

(1) $$\frac{\partial C_{t}}{\partial t}=\frac{\partial }{\partial x}[D\frac{\partial C_{f}}{\partial x}]$$

where *D* is the diffusion coefficient of chloride ions in concrete (m^2^·s^−1^); *x* is the depth from the concrete erosion surface. Ct is the total chloride ion concentration at the distance, which can be represented as Ct = Cf + Cb; Cf and Cb are the free and binding chloride ion concentration in concrete, respectively; R was always used to describe the binding capacity of chloride ion in concrete, which can be represented as R = Cf/Ct, according to the test method recommended in [16]; R = 0.85 can be achieved in this experiment.

Under the test conditions described in this study, the initial condition of the above equation is that the initial chloride ion concentration inside the concrete is $C\left(x>0,t=0\right)$; the boundary condition is that the chloride ion concentration on the concrete surface is $C\left(x=0,t>0\right)=C_{s}$. The analytical solution of the above Formula (1) can be obtained as below:

(2) $$C_{f}\left(x,t\right)=C_{0}+\left(C_{s}-C_{0}\right)[1-erf(\frac{x}{2\sqrt{RD_{a}t}})]$$

where $C_{f}\left(x,t\right)$ is the concentration of free chloride ions at the depth *x* from the surface at time t. erf is the error function: $\mathrm{erf}\left(z\right)=\frac{2}{\sqrt{\pi }}\int_{0}^{z}exp(-\beta^{2})d\beta$; Da is the apparent chloride ion diffusion coefficient (m^2^/s) that characterizes the concrete during the entire immersion period.

**Figure 8:**
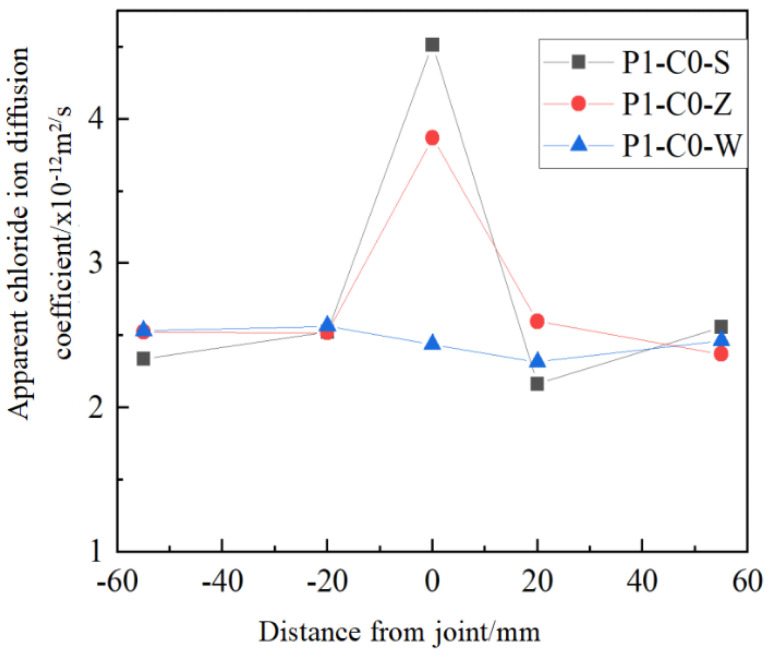
Transverse distribution of apparent chloride diffusion coefficient of bending specimens with different joint types.

According to Formula (2), the chloride ion concentration at various sections of pre-compressed jointed concrete after 300 days of soaking was fitted to determine the apparent chloride ion diffusion coefficient Da at different distances from the joint. Figure 8 presents the apparent chloride ion diffusion coefficients, derived from the fittings, at various distances from the joint. The findings show that for concrete specimens with wet joints and chiseled wet joints, the apparent chloride ion diffusion coefficient at the joint is higher compared to the areas on either side of the joint, suggesting that the cement mortar accumulated at the joint accelerates chloride ion transmission. Additionally, the apparent chloride ion diffusion coefficients at the joints for concrete specimens with direct wet joints and chiseled wet joints increased by 85.31% and 58.75%, respectively, compared to jointless concrete specimens. This suggests that roughening the joint surface can reduce the chloride ion erosion rate at the joints, thereby mitigating the adverse effects on the durability of concrete structures.

### 3.2. Influence of Bending Load Level on Chloride Ion Transport in Bending Specimens

**Figure 9:**
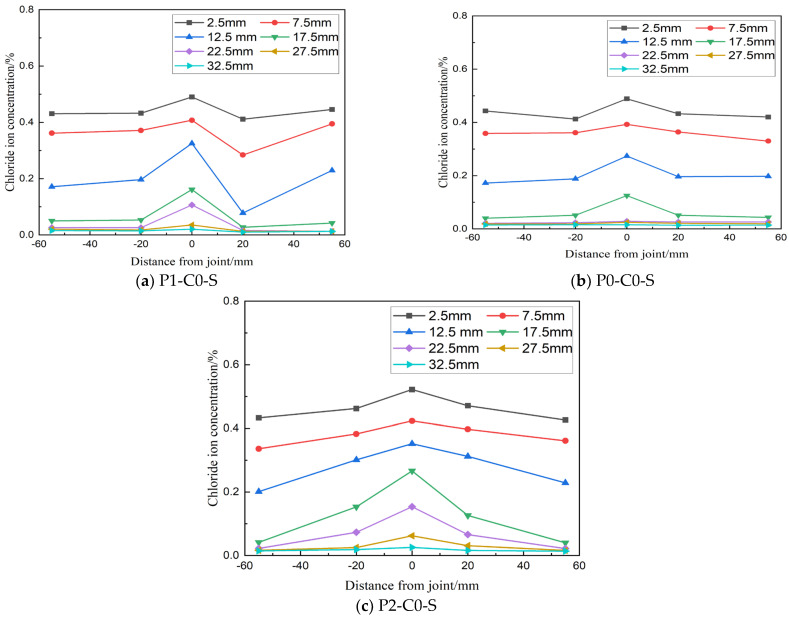
Transverse distribution of chloride ion concentration in bending specimens with different load levels.

The lateral distribution of chloride ion concentration in load-bearing beams under different load levels is illustrated in Figure 9. At load levels P0 and P1, there are no significant cracks in the concrete, and chloride ion transmission is primarily influenced by the joint, with minimal effect observed at distances from the joint (±20 mm and ±55 mm), as shown in Figure 9a,b. However, at load level P2, a crack with a width of 0.095 mm appears at the joint, creating a new pathway for chloride ion transmission. This promotes pronounced lateral diffusion near the joint, causing a significant increase in chloride ion concentration at 20 mm on both sides of the joint compared to farther distances, as depicted in Figure 9c.

#### 3.2.1. Chloride Ion Distribution at Joint Section

**Figure 10:**
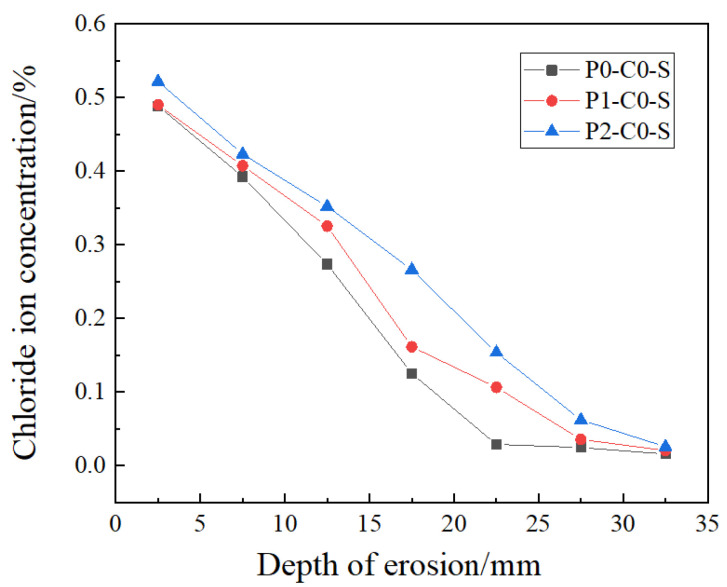
Profiles of chloride ion concentration at concrete joint with different load levels.

To better understand how load levels affect chloride ion transmission at the joints of load-bearing beams, we analyzed chloride ion concentrations in specimens subjected to different load levels, but with the same mix ratio (JZ) and joint type (direct wet joint). Figure 10 illustrates the chloride ion distribution at the joint.

The results indicate that chloride ion concentrations are lower in specimens with load level P0 compared to those with load levels P1 and P2. Notably, at an erosion depth of 7.5 mm, chloride ion concentrations in specimens with load levels P2 and P1 are 1.037 and 1.078 times higher, respectively, than those in specimens with no applied load. At an erosion depth of 17.5 mm, concentrations for load levels P1 and P2 are 1.29 and 2.13 times higher, respectively, compared to the unloaded specimens.

For load level P1, the chloride ion concentration at the joints is lower than in the P2 specimen but significantly higher than in the unloaded specimen. This increase in concentration may be due to micro-cracks and other damage within the joints that occur during loading, even at lower load levels, thereby accelerating chloride ion transmission.

#### 3.2.2. Bending Load Levels on Chloride Ion Diffusion Coefficient

**Figure 11:**
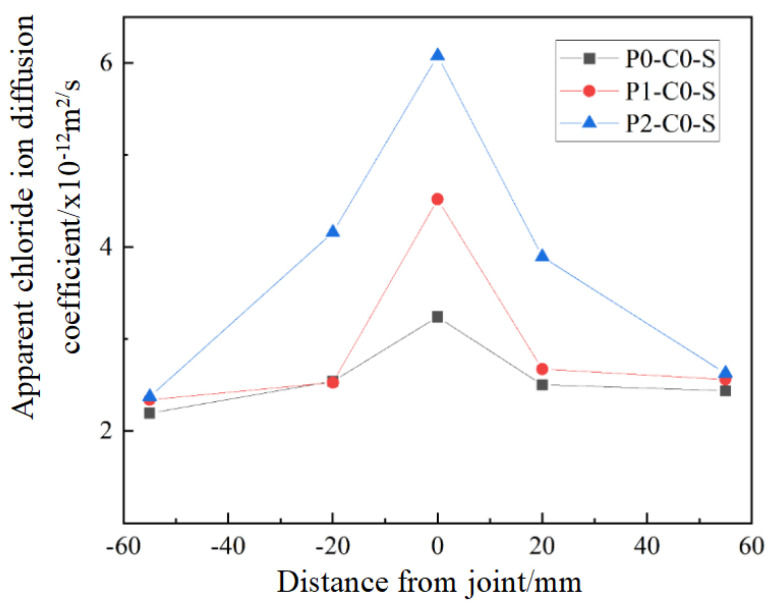
Transverse distribution of apparent chloride ion diffusion coefficient of flexural specimens under different load levels.

Figure 11 illustrates the lateral distribution of the apparent chloride ion diffusion coefficients in concrete specimens subjected to different load levels. The findings indicate that at the joint position, the apparent chloride ion diffusion coefficients increase with load levels, where specimens under load levels P1 and P2 show a 39.5% and 87.7% increase, respectively, compared to P0 (no load). This indicates that elevated bending loads contribute to more extensive joint damage, raising the diffusion coefficient. At 20 mm from the joint, the diffusion coefficient for specimens under load level P2 is lower than at the joint but higher than for P0 and P1, indicating that the pore structure near the joint has been altered under the cracking load (Pcr). However, at 55 mm from the joint, the diffusion coefficients for different load levels are nearly identical, suggesting that chloride ion transmission is not significantly affected by the joint at this distance.

### 3.3. Effect of CCCW Content on Chloride Ion Transport in Bending Specimens

(1) Chloride ion concentration along the lateral distribution

**Figure 12:**
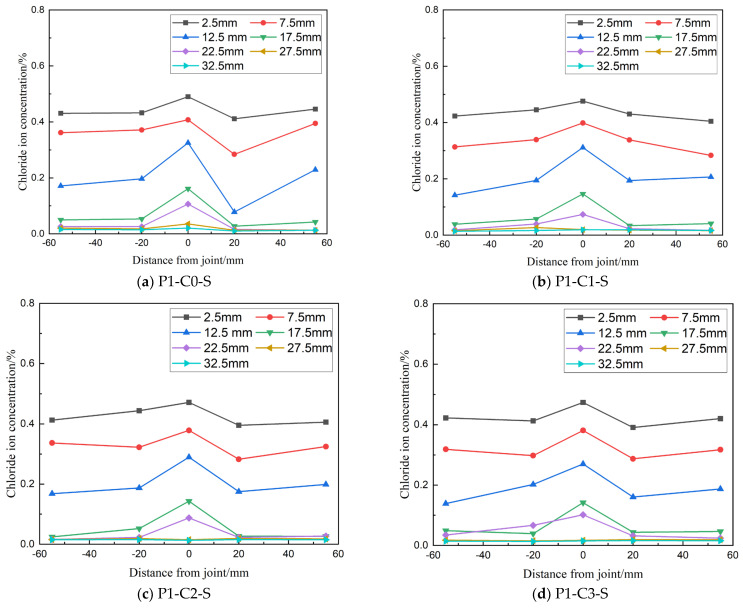
Transverse distribution of chloride ion concentration in bending specimens with different CCCW content.

Figure 12 illustrates the lateral distribution of chloride ion concentration in flexural specimens with varying dosages of Cementitious Capillary Crystalline Waterproofing (CCCW). The key observations show that in specimens with different CCCW dosages, chloride ion concentrations at the joints are consistently higher than at positions farther away, with concentrations decreasing as the distance from the joint increases. Specifically, for CCCW dosages of 1%, 2%, and 3%, the chloride ion concentration at a depth of 0–20 mm at the joint is 1.44, 1.35, and 1.33 times higher, respectively, than at 55 mm away from the joint, indicating that joints negatively affect concrete’s resistance to chloride ion erosion. Additionally, within the 0–20 mm depth range at the joint, free chloride ion concentrations decrease with increasing CCCW dosage. For concrete with 1%, 2%, and 3% CCCW, chloride ion concentrations are 3.9%, 7.9%, and 9.3% lower, respectively, compared to concrete without CCCW (P1-C0-S). This suggests that higher CCCW content enhances the concrete’s resistance to chloride ion erosion by mitigating the adverse effects of bending loads on joint integrity.

#### CCCW Content on Apparent Chloride Ion Diffusion Coefficient

**Figure 13:**
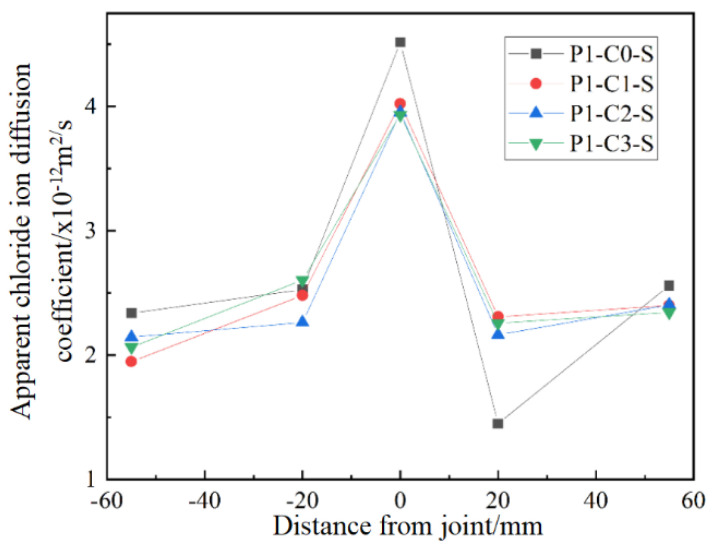
Transverse distribution of apparent chloride ion diffusion coefficient of bending specimens with different CCCW content.

Figure 13 illustrates the apparent chloride ion diffusion coefficients for concrete specimens with varying CCCW contents. The key observations indicate that the apparent chloride ion diffusion coefficients at the joints are consistently higher than those at positions away from the joints, demonstrating that chloride ion erosion is most pronounced at the joints. Concrete specimens containing CCCW display reduced apparent chloride ion diffusion coefficients at the joints compared to those without the additive. Specifically, with a CCCW dosage of around 1%, the apparent diffusion coefficient at the joints is approximately 10% lower than in specimens without CCCW. This suggests that a CCCW dosage of about 1% enhances joint resistance to chloride ion erosion, although further increases in CCCW dosage do not provide significant additional benefits.

## 4. Conclusions

This study investigates the impact of bending load levels and joint types on chloride ion concentration and diffusion in jointed concrete specimens. Experiments were conducted with varying bending load and joint configurations to evaluate these effects. Under identical bending load levels, the chloride ion concentration and apparent chloride ion diffusion coefficient at concrete joints were consistently higher than at chiseled joints.

In summary, direct-wet construction joints under a sustained bending load of 0.6 P_n_ increase the apparent chloride diffusion coefficient (D_a_) at the interface by approximately 50%. Incorporating 1% cementitious capillary crystalline waterproofing reduces that joint-specific D_a_ by about 10% and lowers the peak surface chloride concentration by roughly 8%. Based on these results, we recommend a minimum 1% CCCW dosage for coastal concrete members with joints subjected to flexural loads to meaningfully enhance their resistance to chloride ingress. This observation underscores the importance of incorporating chiseling as an effective strategy to reduce chloride ion penetration and improve durability at joints. Chiseling appears to mitigate the adverse effects of chloride ion attack by enhancing the surface bonding between concrete segments.

Future work will subject jointed specimens with and without 1% CCCW to standard fire exposure (ISO 834 temperature time curve [42]) and rapid cooling, following the methodology of *EN 1992-1-2:2004* [43] (design of concrete structures for fire resistance; https://doi.org/10.1016/j.jmrt.2020.03.015), to assess any synergistic degradation in microstructure and permeability.

## Figures and Tables

**Table 1:** The cement composition in Table 1 is in line with typical OPC values reported in Taylor (1997) [29].

Element	MgO	SiO_2_	SO_3_	CaO	K_2_O	Al_2_O_3_	Na_2_O	Fe_2_O_3_	LOI
Content (%)	1.2	21.6	1.6	65.8	0.7	4.3	0.4	2.6	1.8

**Table 2:** Concrete mix ratio (kg/m^3^) [30].

Serial Number	Cement	Water	Sand	Stone	Superplasticizer	CCCW
C0	413	157	736	1104	6.2	0
C1	408.87	157	736	1104	6.2	4.13
C2	404.74	157	736	1104	6.2	8.26
C3	400.61	157	736	1104	6.2	12.39

**Table 3:** Experimental parameters of concrete specimens [37,38].

Parameters	Specimen No.	Load Level	CCCW Content	Joint Form	
Joint form	P1-C0-S	0.6P	0	Smooth wet joint	
	P1-C0-W	0.6P	0	No joint	
	P1-C0-Z	0.6P	0	Roughened wet joint	
Load level	P1-C0-S	0.6P	0	Smooth wet joint	
	P0-C0-S	0	0	Smooth wet joint	
	P2-C0-S	P	0	Smooth wet joint	
CCCW content	P1-C0-S	0.6P	0	Smooth wet joint	
	P1-C1-S	0.6P	1%	Smooth wet joint	
	P1-C2-S	0.6P	2%	Smooth wet joint	
	P1-C3-S	0.6P	3%	Smooth wet joint	

**Table 4:** Loading strains of different specimens with flexural load.

Specimen No.	Average Strain Value of Steel Bar/ (×10^−6^)	Crack Width/Location /mm
P1-C0-S	90	0
P1-C0-W	86	0
P1-C0-Z	82	0
P0-C0-S	0	0
P2-C0-S	155	0.095 (at the seam)
P1-C1-S	86	0
P1-C2-S	85	0
P1-C3-S	80	0

## Data Availability

The original contributions presented in this study are included in the article. Further inquiries can be directed to the corresponding author.
